# Vaginal cuff dehiscence with bowel evisceration after robotic hysterectomy

**DOI:** 10.4274/tjod.47640

**Published:** 2014-12-15

**Authors:** Ali Akdemir, Enes Taylan, Ahmet Mete Ergenoğlu, Ahmet Özgür Yeniel, Fatih Şendağ, Mehmet Kemal Öztekin

**Affiliations:** 1 Ege University Faculty of Medicine, Department of Obstetrics and Gynecology, İzmir, Turkey

**Keywords:** Robotic hysterectomy, vaginal cuff dehiscence, bowel evisceration

## Abstract

Vaginal cuff dehisence with bowel evisceration after hysterectomy is a very rare complication. However, the incidance of this complication appears to be increased with the widely used techniques of laparoscopic surgery especially with robotic hysterectomy. In this case report we aimed to evaluate the risk factors and treatment methods for this complication.

## INTRODUCTION

Vaginal cuff dehiscence is defined as partially or totally seperation of anterior and posterior vaginal cuff layers. Vaginal cuff dehiscence with bowel evisceration is a rare complication after hysterectomy, however, as laparoscopic and especially robotic surgery becomes widely performed around the world the incidence of this complication increased more^(1,2,3,4)^. In a study which investigated the incidence of vaginal cuff dehiscence after total abdominal hysterectomy, vaginal hysterectomy and laparoscopic hysterectomy reported the incidence of vaginal cuff dehiscence as 0.12-0.99%, 0.12-0.29% and 0.47-4.93% respectively^(2)^.

In this case report we aimed to investigate the relation and risk factors of vaginal cuff dehiscence after robotic hysterectomy which is a very rare complication.

## CASE

**Figure 1:**
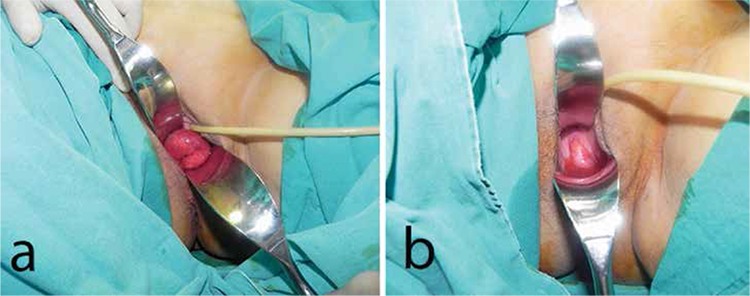
a) Eviscerated intestinal loops seen through the vagina b) Vaginal cuff dehiscence seen after spontaneous regression of intestinal loops

Fifty-five year old woman with the diagnosis of endometrial cancer (endometrioid adenocarcinoma grade 1) hospitalized, and robotic hysterectomy and bilateral salpingooferectomy planned. After the operation pathological result reported as tumor was limited to endometrium and patient was decided not have additional chemotherapy or radiotherapy. Almost 6 months after surgery patient admitted to our emergency department with severe pelvic pain started after sexual intercourse, and patient immediately refered to our clinic due to vaginal cuff dehiscence with bowel evisceration revealed by pelvic examination. At the peroperative vaginal examination we observed small intestinal loops passed through the vaginal cuff into the vagina, however, patient had no clinical or biochemical sign of peritonitis. The assesment of eviscerated intestinal tissue with consultation of a general surgeon resulted as it is viable (Figure 1). During the vaginal inspection under general anaesthesia intestinal loops spontaneously entered back to the abdominal cavity and a 4 cm total dehiscence at the vaginal cuff appeared. After removal of the debris on the edges of the vaginal cuff layers the defect closed with 0 PDS via vaginal approach. Later on a perneous drain tube inserted through the vaginal cuff into the pelvic cavity and the procedure ended without any complication. At the end of postoperative first day the drain tube taken out and the next day patient discharged from the hospital. Patient called for control 3 and 6 months later, and she described no more complaint and had a normal daily life.

## DISCUSSION

Although vaginal cuff dehiscence after hysterectomy is a rare complication of abdominal approach, by the widely use of laparoscopic techniques the incidence of this complication has reported with an increased rate from many centers. A considerable amount of study showed that complication is more common especially after robotic surgery^(1,2,3,4,5)^.

In a comprehensive review of the literature published by Cronin et al., a two-layered closure of vaginal cuff, preferring monopolar electrocautery to bipolar and the use of bidirectionel barbed suture suggested as may decrease the risk of cuff dehiscence^(4)^. Besides, the choice of cuff repair is affected by many factors such as clinical stability of the patient, whether a bowel evisceration is present, the presence of ischemic or damaged tissue, the need for additional surgical procedures.

Kho et al. reported 21 cases of vaginal cuff dehiscence in their study which included 510 patients with an incidence of 4.1%. The most common triggering factor in these patients was defined as intercourse and the mean interval to dehiscence was reported as about 43 days. The most common sypmtoms found were vaginal bleeding and watery discharge^(5)^.

Different explanations were suggested for the reasons of vaginal cuff dehiscence after robotic hysterectomy. Possible factors for this complication may be intercourse, increased intraabdominal pressure, vaginal trauma, connective tissue diseases, corticosteroid use or immune supression, smoking, higher Body Mass Index (BMI) and malignancy. However, many studies proposed the surgical technique as the main factor. Specifically the thermal injury and the suturation technique are proposed to increase the risk of cuff dehiscence after laparoscopic and robotic hysterectomy^(6,7,8)^.

Although the main goal for laparoscopic hysterectomy is to perform all steps by laparoscopic approach, Ucella et al. reported that transvaginal closure of vaginal cuff is more simple, faster and has 3-9 fold less risk for cuff dehiscence^(9)^.

The increased risk of cuff dehiscence in oncological cases is probably related to the previous chemotherapy or radiotherapy treatments, age, malnutrition, postoperative complications such as infection and hematoma^(1)^.

Muffly et al. demonstrated that sutures tied with robotic approach can be untied by a force of 57.4 N, however, a manually tied suture needs 112.2 N force to be untied. Also using polypropylene suture during robotic surgery may have better results^(8)^.

Management of patients with vaginal cuff dehiscence includes hospitalization, intravenous volume replacement, wide spectrum antibiotherapy, and cuff repair in 24 hours^(1,4)^. Vajinal cuff can be repaired via vaginal, abdominal and laparoscopic approaches. There is no current evidence that suggests one approach is preferred to others. Many factors such as clinical condition of the patient, signs of peritonitis, and the viability of eviscerated bowels have more importance for the decision of repair technique. In a clinically stable patient and without any additional complication vaginally repair of cuff is suggested^(4)^.

In our case, patient admitted to our clinic after a long duration almost 6 months and the triggering factor detected as intercourse. Despite the history of endometrial cancer diagnosis, she did not need radiotherapy or chemotherapy treatments. While the evaluation of the surgical video record of the robotic hysterectomy we learned that vaginal cuff sutured one layered and continously, and during the closure of vaginal cuff insufficent amount of tissue used. These factors obviously increased the risk of vaginal cuff dehiscence as mentioned above by many studies. Unfortunately, besides many advantages of magnified view in robotic surgery it may also trick surgeon to use an insufficent amount of tissue during cuff closure^(10)^.

As a conclusion, vaginal cuff dehiscence is a rare but very serious complication which may occur after any mode of hysterectomy. As its incidence increases more researches need to be done to prevent this complication and also reveal its etiology.
